# Coalescence of Binary Droplets in the Transformer Oil Based on Small Amounts of Polymer: Effects of Initial Droplet Diameter and Collision Parameter

**DOI:** 10.3390/polym12092054

**Published:** 2020-09-09

**Authors:** Yiting Wang, Lijuan Qian, Zhongli Chen, Fang Zhou

**Affiliations:** College of Mechanical and Electrical Engineering, China Jiliang University, Hangzhou 310018, China; P1801085256@cjlu.edu.cn (Y.W.); 14a0104128@cjlu.edu.cn (Z.C.); zhoufang8602@126.com (F.Z.)

**Keywords:** the volume of fluid method, droplet coalescence, off-center collision, droplet diameter, collision parameter

## Abstract

In engineering applications, the coalescence of droplets in the oil phase dominates the efficiency of water-oil separation. To improve the efficiency of water-oil separation, many studies have been devoted to exploring the process of water droplets colliding in the oil phase. In this paper, the volume of fluid (VOF) method is employed to simulate the coalescence of water droplets in the transformer oil based on small amounts of polymer. The influences of the initial diameter and collision parameter of two equal droplets on droplet deformation and coalescence time are investigated. The time evolution curves of the dimensionless maximum deformation diameter of the droplets indicate that the larger the droplet diameter, the more obvious the deformation from central collisions. As the collision parameter increases, the contact area of the two droplets, as well as the kinetic energy that is converted into surface energy, decreases, resulting in an increase in droplet deformation. Furthermore, the effects of the initial droplet diameter and collision parameter on coalescence time are also investigated and discussed. The results reveal that as the initial droplet diameter and collision parameter increase, the droplet coalescence time increases.

## 1. Introduction

Collision study of liquid droplets has been widely used in the nature, aerospace, industrial, biomedical, and other fields, with respect to aspects such as polluted air and global atmosphere [1], fuel-burning [2], polymer-solvent spray [3], the petroleum industry [4], and 3D printing [5]. In thermal engineering, transformer oil generally works at a temperature of 60–80 °C, and long-term contact with metals will cause the oil to oxidize [6] and generate a small amount of water and polymer, which will cause poor heat dissipation and safety problems. Therefore, it is very necessary to remove water from the transformer oil.

Kang et al. [7] used a stability analyzer to explore the effects of demulsifiers on the demulsification process of emulsions, and indicated that the coalescence of the dispersed phase droplets is the crucial step of the oil-water separation. Angeli et al. proposed that the coalescence of two dispersed droplets in a turbulent flow field would require the two droplets to collide and then interact until the liquid film between the droplets is reduced to below a critical liquid film rupture thickness [8]. Liao et al. also reached a similar consclusion to that of Angeli et al., proposing that the droplets will coalesce when the interaction time of the dispersed droplets is greater than the time to reduce the liquid film to below the critical liquid film thickness [9].

The coalescence of water droplets in the oil phase has been described by a few investigators. In the case of liquid–liquid two-phase droplet collision, the shape and size of the droplets, the distance between the droplets, the collision parameter and the droplet velocity will affect the droplet collision results [10]. Some experiments have been carried out on the collision and coalescence of dispersed droplets in oil. Using a microfluidic device, droplet collision experiments were conducted in flowing oil-in-water by Krebs et al. [11]. They showed that lower capillary number and oil phase viscosity would reduce droplet coalescence time. Huang et al. [12] experimentally studied the effect of Alkali/Surfactant/Polymer (ASP) on the efficiency of oil-water separation and proved that the addition of demulsifier achieves a good treatment effect on oil-water separation. Moreover, coalescence time models of different water-oil mixing were obtained. Dudek et al. [13] proposed a new microfluidic method for investigating the effects of droplet size and approach velocity of the colliding droplets. The results indicated that the larger the approach velocity of the droplet, the shorter the collision time and coalescence time. He et al. experimentally studied the coalescence and sedimentation behavior of iron droplets based on a mechanical stirring method. The results showed that although mechanical agitation increased the collision probability of the iron droplets, the non-center collision and coalescence of droplets became difficult due to the increase in the relative velocity of the iron droplets [14]. 

Numerical simulations could provide valuable insight into the mechanism of coalescence of binary droplets. The simulation results are validated by comparing the droplets’ behaviors with the experimental data, confirming that the numerical simulations are capable of predicting the coalescence of droplets with high accuracy. At the same time, numerical simulation can provide flow field diagrams and theoretical analysis to obtain more valuable mechanism research. Mansouri et al. [15] used the volume of fluid (VOF) method and the level-set (LS) method to simulate the coalescence of two droplets in the oil phase, and the effects of droplet velocity, water-oil interfacial tension, and the viscosity of the continuous phase on coalescence time were investigated. The results revealed that larger droplet velocities led to shorter coalescence time, which is similar to the conclusion of Yuan et al. [16]. Mohammadi et al. [17] considered the coalescence of two water droplets in oil. They also reached a similar conclusion to Mansouri’s, that higher oil phase viscosity or lower water-oil interfacial tension will promote droplet coalescence [15]. Mino et al. used the lattice Boltzmann method (LBM) to numerically study the coalescence of droplets in an oil-in-water emulsion. The simulation results demonstrated that the emulsion will form droplets of larger diameter through a filter with larger pore spacing, but it is difficult for the larger-diameter droplets to coalesce; thus, it is difficult to separate water and oil [18]. Bresciani et al. [19] investigated the effect of the electric field on the coalescence mechanism of droplets, and a mathematical model was established to calculate the collision velocity and the coalescence time of the droplet. The results indicated that the coalescence of droplets could be accelerated by changing operating parameters such as temperature and electric field. The investigation of the size distribution of liquid droplets and droplet coalescence stability is of high importance in water-oil two-phase dispersed flow [20]. Fortelný et al. reviewed the applicability of droplet diameter distribution in the processing of immiscible polymer blends [21]. Dudek et al. proposed that, in the specific oil phase, the droplet diameter distribution increases and the coalescence efficiency decreases [22].

As mentioned above, some studies have been conducted on the effects of physical parameters and control parameters on the coalescence of binary droplets. Furthermore, in the field of the coalescence of water droplets in the oil phase, the different research approaches can roughly be divided into two groups. On the one hand, from a driving method of view, electric fields [23] or coupling of electric and magnetic fields [24] are often used to promote droplet coalescence. On the other hand, some literature focuses on the addition of polymers to promote droplet coalescence, which has recently evolved rapidly, because it can change the water-oil interfacial tension [25]. However, from a microscopic point of view, research into the effect of operating parameters on droplet coalescence is insufficient, such as that into the effects of the initial droplet diameter and collision parameter on droplet deformation and internal flow field. The work presented in this paper selected a representative transformer oil as the continuous phase for numerical simulation and applied the VOF method to track the water-oil interfaces. The accuracy of the numerical model was verified using the experimental data of Qian et al. [26] and the numerical data of Yuan et al. [16]. Then the effects of initial droplet diameter and collision parameter on droplet coalescence were investigated. Time evaluation of the droplet morphology makes it possible to identify collision and coalescence events. From the measured sets of droplet coalescence under each simulated condition, we present the deformation trend and coalescence time distributions. The result can provide a broader theoretical basis for the improvement of water-oil separation technology in the industry, and provide guidance and suggestions for the mechanical design and industrial process of coalescers.

## 2. Numerical Model 

### 2.1. The Governing Equation

The problem studied in this work is the coalescence of binary water droplets in the transformer oil based on small amounts of polymer, which can be described as incompressible, isothermal, immiscible multiphase flow. Among various multiphase flow models, the volume of fluid (VOF) model is considered to be the most suitable one for identifying the water-oil interface [27]. The VOF model introduces an additional transport equation for the volume fraction of the traced fluid, and uses the additional term in the momentum equation to deal with the interface tension forces [28]. In this method, mass and momentum equations can be written as:

(1) $$\frac{\partial \rho }{\partial t}+\nabla \cdot \left(\rho \vec{U}\right)=0$$

(2) $$\frac{\partial \rho \vec{U}}{\partial t}+\nabla \cdot \left(\rho \vec{U}⊗\vec{U}\right)=-\nabla p+\nabla \cdot \left[-\mu \left(\nabla \vec{U}+\nabla {\vec{U}}^{T}\right)\right]+{\vec{F}}_{\sigma }$$

where $\vec{U}$ is the fluid velocity, p is the pressure, $F_{\sigma }$ is the volume surface tension force, and $-\mu (\nabla \vec{U}+\nabla {\vec{U}}^{T})$ is defined as stress tensor.

In the VOF method, α is a volume fraction function varying from 0 to 1, defined as the percentage of the traced fluid’s volume in the volume of the computational grid cell. To track the water-oil interface by solving a single momentum equation through the domain, the VOF model uses a reduced property θ calculated by a volume fraction average of all fluids in the cell, i.e.,

(3) $$\theta =\alpha \theta_{w}+(1-\alpha )\theta_{o}$$

The density ρ and viscosity μ of the water-oil mixture can be obtained by evaluating the following equations:

(4) $$\rho =\alpha \rho_{w}+\left(1-\alpha \right)\rho_{o}\nu$$

(5) $$\mu =\alpha \mu_{w}+\left(1-\alpha \right)\mu_{o}$$

where the subscripts w and o represent the water phase and oil phase, respectively.

### 2.2. Water-Oil Interface Tracking

In this study, the following transport equation is introduced to track and locate the water-oil interface [29]:

(6) $$\frac{\partial \alpha }{\partial t}+\nabla \cdot \left(\alpha \vec{U}\right)=0$$

where the volume function α is defined as the volume fraction of water in each computational cell, i.e., the traced fluid is water in the present study. When a computational grid cell is completely filled with water, the value of α is one; when the cell is filled with oil, α=0; and when there is a water-oil interface in the cell, 0<α<1 [12].

Water-oil interfacial tension makes a pressure jump across the interface for which the gradient is equal to the added volumetric surface force $F_{\sigma }$, the force is applicable only at the interface cell. Equation (7) implies the volumetric surface force $F_{\sigma }$:

(7) $$F_{\sigma }=2\cdot \alpha \cdot \sigma \cdot \kappa \cdot n$$

where σ is the interfacial tension, the surface local curvature κ and the surface normal vector n are defined as:

(8) $$\kappa =\frac{1}{\left|\vec{n}\right|}\left[\left(\frac{\vec{n}}{\left|\vec{n}\right|}\cdot \nabla \right)\left|\vec{n}\right|-\left(\nabla \cdot \vec{n}\right)\right]$$

(9) n=∇α

### 2.3. Computational Setup

**Figure 1:**
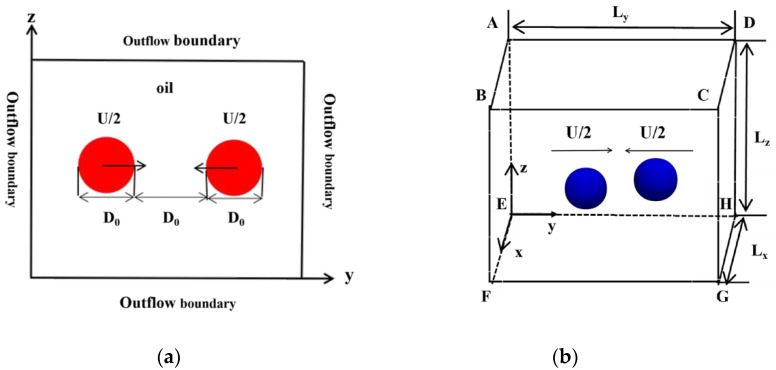
Schematic diagram of the collision calculation domain and initial state of binary equal-sized droplets: (**a**) schematic of the binary droplets center collision; (**b**) schematic of the off-center collision computational domain.

The coalescence of two equal-sized spherical droplets immersed in transformer oil based on small amounts of polymer is considered in the present study. Figure 1 shows the setup of numerical simulation: binary water droplets with the same initial droplet diameters *D_0_* approach and collide with each other with an initial collision velocity *U/2*. The computational domain is a cube and the grid number is 80 × 80 × 80. The same pressure outlet boundary conditions are employed and the effect of gravity is ignored in all calculations.

**Figure 2:**
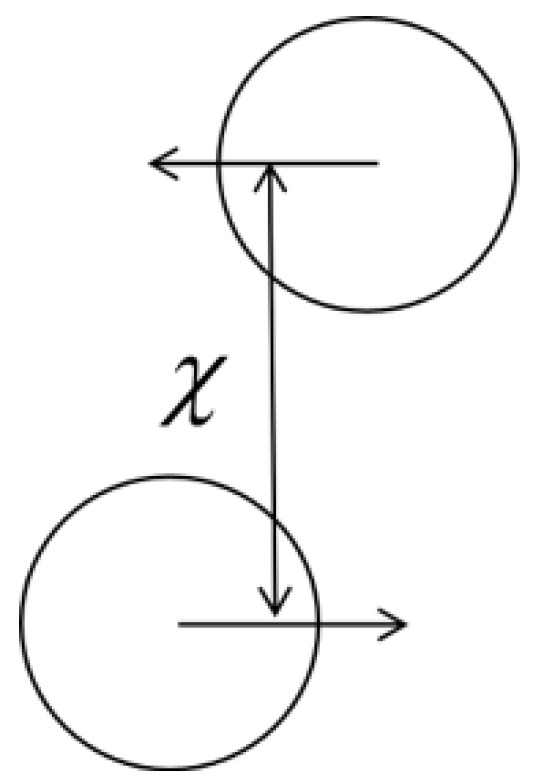
Schematic diagram of the collision parameter.

Table 1 shows the setup of the numerical simulation cases in this study. Three dimensionless parameters are involved: the Weber number (*We*) is defined as the ratio of inertial force to surface tension; the Reynolds number (*Re*) represents the ratio of inertial force to viscous force; the collision parameter (*B*) is defined as the ratio of χ (the projection of the center distance of two droplets in the direction of their relative velocities, as shown in Figure 2) to the initial droplet diameter *D_0_*, and an off-center collision occurs when *B* ≠ 0.

(10) $$We=\rho_{w}D_{0}U^{2}/\sigma$$

(11) $$Re=\rho_{o}D_{0}U/\mu_{o}$$

(12) $$B=\chi /D_{0}$$

Due to the difference in composition and characteristics, the viscosity range of polymer blends is 0.004 to 0.014 Pa·s [30]. Considering ecology, economy and energy efficiency, this work chooses transformer oil with a viscosity of 0.0069 Pa·s as the continuous phase, which is referred to as the oil phase; its density is 963 kg/m^3^. For the water droplets, the density is 1000 kg/m^3^, the viscosity is 0.01 Pa·s. The water-oil interfacial tension σ is 0.0162 kg/s^2^ and the initial collision velocity U is 1.82 m/s.

In all simulations, the following dimensionless time t* is employed,

(13) $$t^{*}=(t-t_{0})(U/2)/D_{0}$$

where $t_{0}$ is the initial time.

## 3. Computing Validation

### 3.1. Grid Independence

**Figure 3:**
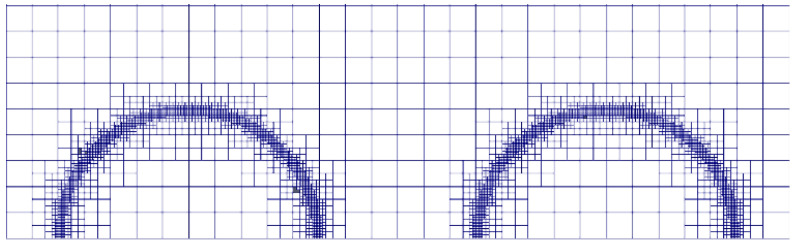
The adaptive mesh generated by interDyMFoam.

The adaptive mesh refinement (AMR) technology can locally refine the two-phase interface region in the computational domain, which reduces the calculation cost and improves the calculation accuracy of numerical simulation [31,32]. Maric et al. [33] developed a dynamic local adaptive mesh refinement algorithm for unstructured meshes in OpenFOAM (OpenFOAM Foundation, OpenFOAM 5.0, Delaware, USA) to track the liquid-liquid interfaces in two-phase flow. In this paper, the hexahedral adaptive mesh solver interDyMFoam based on the OpenFOAM standard is used to track and locate the water-oil interface. Figure 3 shows the computational mesh generated by interDyMFoam.

**Figure 4:**
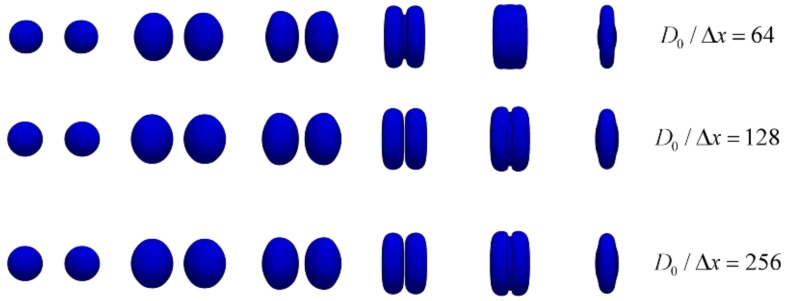
Verification of the grid independence under three different grid resolutions.

Figure 4 shows the morphological evolution of coalescence of two equal-sized droplets under three different grid resolutions, Δx is the minimum mesh size.

It can be observed that in the early stages of the collision process, the droplet shapes are similar for different mesh resolutions. In the later stages of the collision process, when the grid resolution is $D_{0}/\Delta x=64$, the collision time of droplets is advanced, and the droplet morphology changes. When the grid resolution $D_{0}/\Delta x=128$ and $D_{0}/\Delta x=256$, the droplets also have the same morphology. To increase computational efficiency and save calculation time, the grid resolution $D_{0}/\Delta x=128$ is used for all calculations.

### 3.2. Model Validation

**Figure 5:**
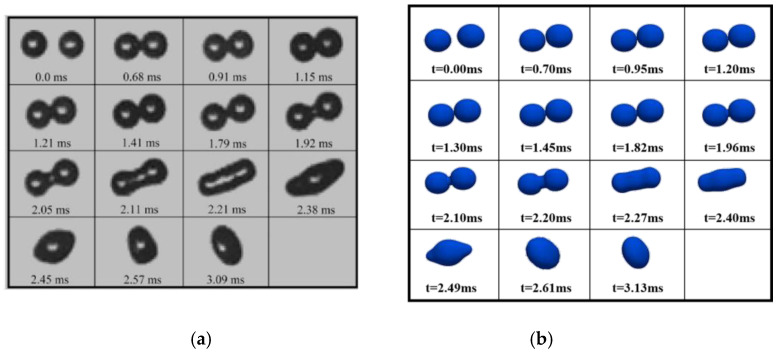
Comparison with the experimental images of Qian et al. [26]: (**a**) the experiment result; (**b**) the simulation result.

**Figure 6:**
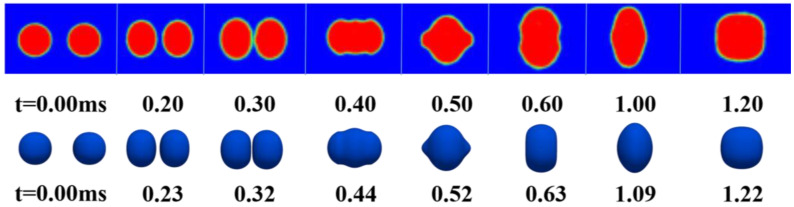
Numerical verification of water droplet collision in the oil phase: the simulation results of Yuan [16] (**top**); the numerical simulation results of this model (**bottom**).

Figure 5 and Figure 6 show the droplet shapes predicted by the present study together with the experimental and numerical results provided by Qian et al. [26] and Yuan et al. [16], respectively. These figures imply that under the same conditions, the numerical results of the present study are in good agreement with those in the literature, which indicates that the model in the present study is qualified to simulate the coalescence of water droplets in the transformer oil based on small amounts of polymer.

## 4. Results and Discussion

In the water-oil separation process, the initial diameter of the droplets changes with the physical and chemical properties of the liquid and the device settings. Therefore, in most precipitation separation processes through the orifice plate, the control of the droplet diameter is very important. Moreover, in the water-oil polymer blend, the distribution of droplets will affect the collision parameter of the droplets, and further affect the efficiency of water-oil separation. Therefore, this work focuses on the effects of droplet diameter and collision parameter on droplet coalescence.

### 4.1. The Effect of Initial Droplet Diameter (D_0_) on Droplet coalescence

**Figure 7:**
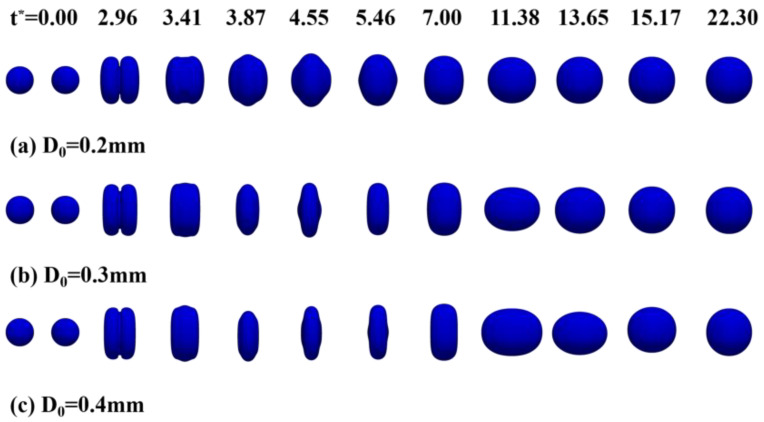
Time evolution of two water droplets coalescence in the oil phase under different initial droplet diameters (**a**) D_0_ = 0.2 mm, (**b**) D_0_ = 0.3 mm, (**c**) D_0_ = 0.4 mm.

To study the effect of the initial droplet diameter (*D_0_*) on coalescence, three simulations were carried out with initial droplet diameters of 0.2 mm, 0.3 mm, and 0.4 mm at the same collision velocity. Figure 7 shows snapshots of the droplet coalescence of these simulations.

As shown in Figure 7, the droplets approach each other in the oil phase, collide, coalesce and eventually form a larger spherical droplet. When *D_0_* = 0.4 mm, the droplets collide and form a connected interface (t* = 2.96). However, the interfaces between the two droplets are different due to different droplet diameters. At t* = 3.41, the interface disappears and the droplet deforms into a thick flat shape. Then, the droplet begins to expand radially and shrink axially. When t* = 7.00, a pressure difference is generated on both sides of the droplet due to the effect of interfacial tension, and the droplet expands axially. When t* = 13.65, the droplet shrinks axially. In the later period of collision, the oscillation behavior caused by surface tension gradually dampens and the droplet finally recovers into a larger spherical droplet (t* = 22.30).

**Figure 8:**
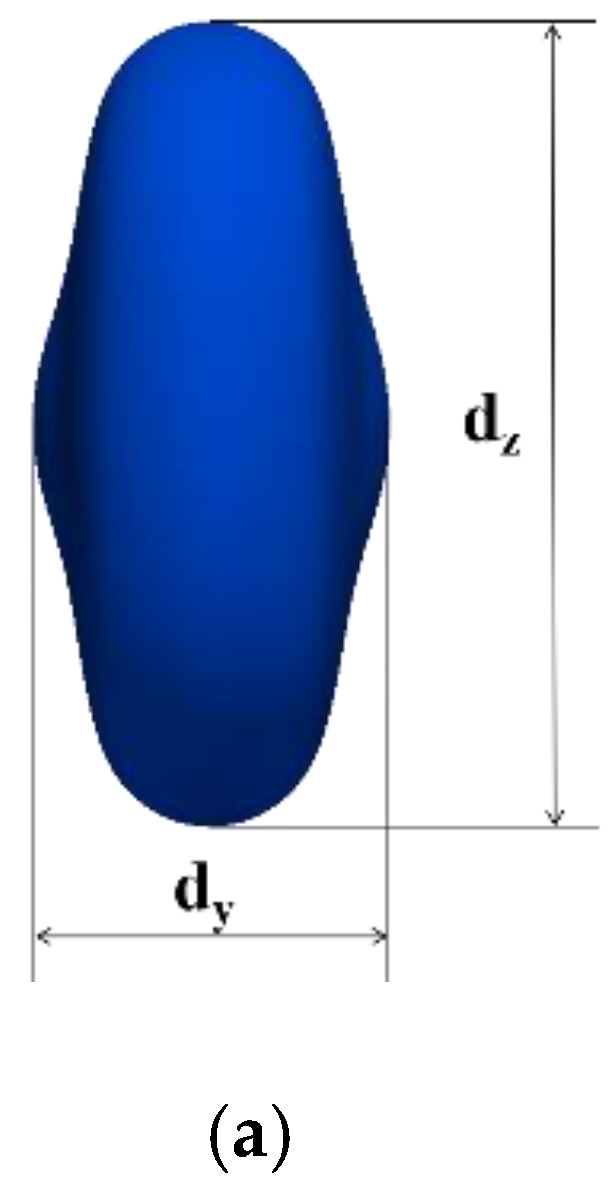

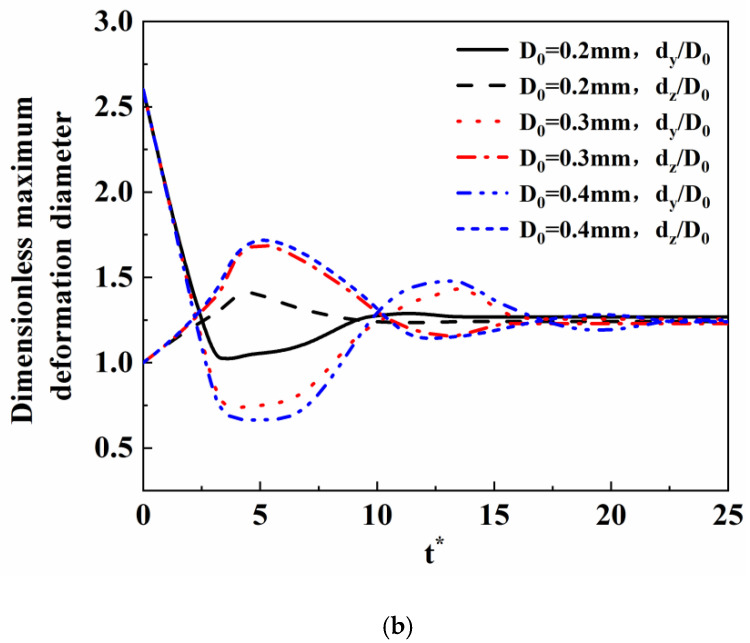
(**a**) The droplet deformation parameters; (**b**) the dimensionless time evolution of the dimensionless maximum deformation diameter of droplets with different initial droplet diameters.

Figure 8a shows the deformation parameters during the droplet collision, where *d_y_* is the maximum axial diameter and *d_z_* is the maximum radial diameter. In this paper, dimensionless maximum deformation parameters *d_y_/D_0_* and *d_z_/D_0_* are used to describe the droplet deformation. Figure 8b shows the evolution of the dimensionless maximum deformation diameter of three different initial droplet diameters with the dimensionless time.

It can be seen from Figure 8b that the evolution processes of collisional complexes for different initial droplet diameters are approximately the same. After the two droplets approach each other and collide to form a larger droplet, they begin to expand radially until they reach the maximum radial size. The droplet then begins to shrink radially and expand axially due to the effect of surface tension. As shown in Figure 8b, as the initial droplet diameter decreases, the deformation curve tends to stabilize earlier. According to the law of conservation of mass, the final spherical droplet diameter is larger than the initial droplet diameter, and the stable value of the dimensionless maximum deformation curve is above 1.

### 4.2. The Effect of the Collision Parameter (B) on Droplet Coalescence

**Figure 9:**
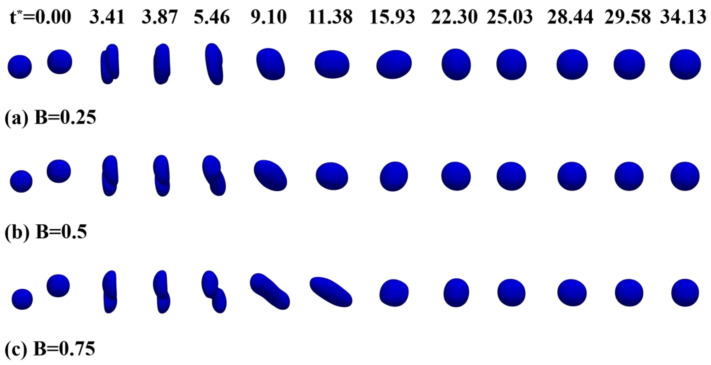
Time evolution of the coalescence of two water droplets in the oil phase under different collision parameters (**a**) B = 0.25, (**b**) B = 0.5, (**c**) B = 0.75.

To investigate the effect of the collision parameter (*B*) on the coalescence of binary droplets in the oil phase, three simulations were executed with collision parameters of 0.25, 0.5, and 0.75, and the numerical results are shown in Figure 9. As the droplets approach each other, the droplet surfaces deform and gradually flatten under the effect of surface tension. It can be observed in Figure 9 that two droplets begin to coalesce and one droplet penetrates into the other one at t* = 3.41. With the development of the coalescence process, the droplet has a significant deformation before t* = 11.38. The droplet then recovers to a nearly spherical droplet under the action of oscillation.

As can be seen from Figure 9, in the early stage of coalescence, the droplet deformation increases with the increase of the collision parameter. When B = 0.25, since most of the kinetic energy of the droplet is converted into surface energy and dissipated energy during the collision process, the surface energy dominates the deformation process and the deformation of the droplet is resisted. Therefore, the droplet will quickly recover to a spherical droplet. This is similar to the conclusion of Alejandro et al. [34], who believed that when the inertial force was too low, the surface tension force played a dominant role and the collision process was conducive to coalescence. When B = 0.5, less kinetic energy is converted into surface energy, so the deformation of the droplet is more obvious than that for B = 0.25. When B = 0.75, only small parts of the droplets collide, and the remaining parts of the droplets are axially stretched at the original speed and direction, and then gradually recover to a spherical shape under the effect of surface tension. Therefore, during the droplet collision process, the maximum axial diameter of the droplet at B = 0.75 is around 127% of the maximum axial diameter of the droplet at B = 0.25.

As can be seen from Figure 7 and Figure 9, the results of coalescence in this paper are significantly different from those of polymer droplets. On the one hand, the settings of the numerical simulation are different. The numerical simulation in this paper focuses on the coalescence of droplets in transformer oil containing a small amount of polymer, which is essentially different from the coalescence of polymer droplets. On the other hand, the deformation degree and mode of the droplet are related to the physical parameters and other control parameters, and any change in parameters will affect the deformation and coalescence mode of the droplets. Therefore, the results of coalescence in this paper are different from the results of polymer droplet coalescence.

**Figure 10:**
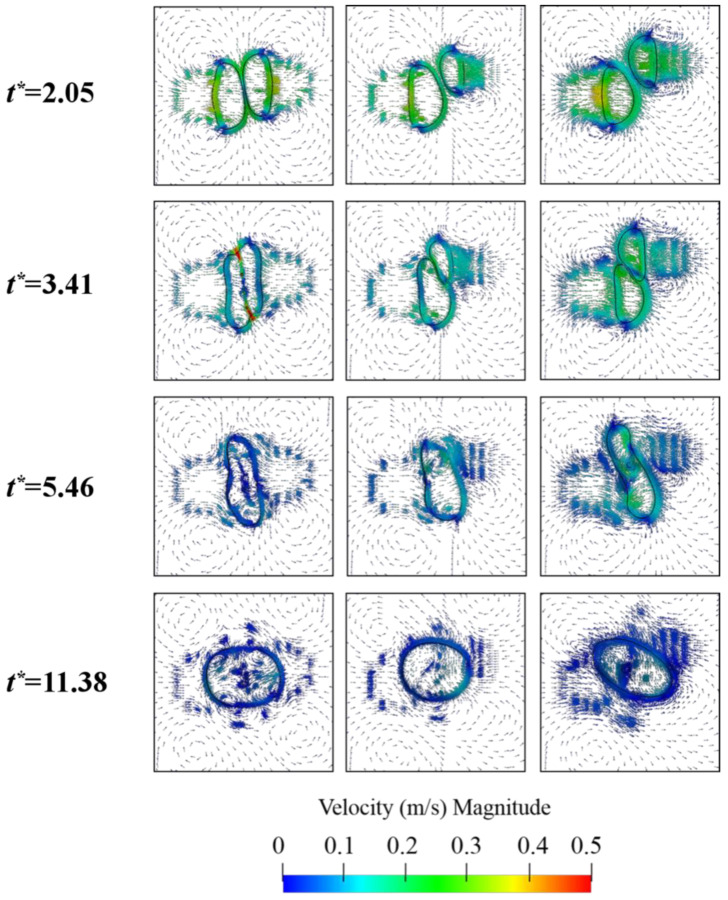
Evolution of the velocity field of the droplet coalescence process under different collision parameters. From left to right: B = 0.25, B = 0.5, B = 0.75.

To further explore the effect of the collision parameter on the droplet collision, the velocity field of the droplet coalescence process is shown in Figure 10. The velocity vector arrows are used to describe the movement of the flow field, and the velocity vector color is used to describe the velocity magnitude.

When the droplets approach each other, there is an increment of the pressure between them, and therefore the velocity of the droplet increases (t* = 2.05). Inside the droplet with B = 0.25, the velocity remains at around 0.1 m/s (t* = 3.41), while in the local region of the droplets contact, the droplet velocity increases to a value of 0.5 m/s due to the rapid increase in pressure in this region. For droplets with B = 0.5 and B = 0.75, due to the viscosity of the oil phase, the velocity of the droplets decreases at t* = 3.41. As the collision parameter increases, only a small part of the kinetic energy of the droplet is converted into surface energy, and the remaining kinetic energy is used for the droplet collision. Therefore, when t* = 5.46, the internal velocity of the droplet with B = 0.75 is the largest. In addition, it can be seen from Figure 10 that, when B = 0.75, the velocity vectors on both sides of the droplet are denser, and the streamlines explain the stretch deformation of the droplet in the coalescence deformation stage. According to the research by Jiang et al. [35], in the process of the droplet recovering to a spherical shape, the internal pressure of the droplet increases under the action of surface tension. Therefore, the velocity inside the droplet flows toward the center of the droplet, Figure 10, t* = 11.38.

### 4.3. The Coalescence Time 

**Figure 11:**
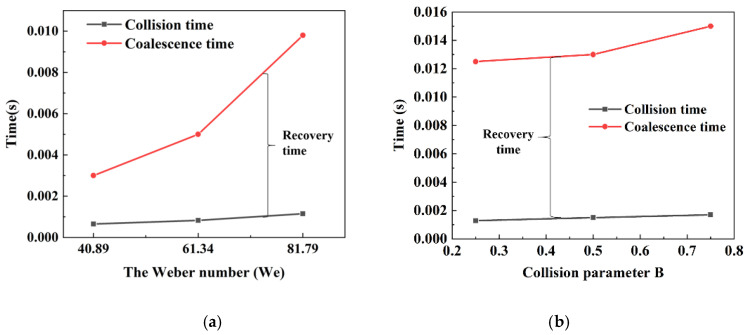
The collision time, coalescence time and recovery time evolution of water droplet collision in the oil phase: (**a**) different Weber numbers; (**b**) different collision parameters.

After the droplets collide, coalescence, bounce, stretch separation or reflection separation may occur [26]. Coalescence time is an important parameter for indicating the ease or complexity of droplet coalescence. However, previous studies haven’t provided a precise definition of coalescence time. The coalescence time defined by Mohammad et al. is the time required for two droplets with the same diameter to move toward each other until the film between the droplets is drained out [17]. The coalescence time is defined by Krebs et al. as the time required for the distance between droplets to reach the coalescence critical thickness *h_c_* [11]. However, these two definitions are not accurate; on the one hand, if two droplets collide at high speed, they may break up after coalescence, and the result of this collision cannot be considered coalescence, while on the other hand, the definition of *h_c_* has not been unified. Therefore, the coalescence time in this study is defined as the time required for two droplets of equal diameter to collide with each other, coalesce and recover to a nearly spherical droplet. According to the definition, the droplet coalescence time is divided into two parts, one is the droplet collision time, that is, the time required for the two droplets approach each other until they first touch, and the other is the recovery time, that is, the time required for the collision complex to recover into an approximately spherical droplet. The evolution of the collision time, recovery time, and coalescence time of droplets with Weber numbers of 40.89, 61.34, and 81.79 (the corresponding initial droplet diameters are 0.2 mm, 0.3 mm, and 0.4 mm, respectively) and collision parameters of 0.25, 0.5, and 0.75 are shown in Figure 11a,b. The two curves represent the collision time and coalescence time of the droplet, respectively, and the difference between the two curves is the recovery time. 

As shown in Figure 11a, the droplet collision time, coalescence time and recovery time increase with the increase of the Weber number (*We*). In this paper, the ratio of the distance between two droplets to the initial droplet diameter is fixed at 1. The collision time is proportional to the distance between the droplets. In addition, the Weber number (*We*) increases with the increase of the initial droplet diameter. Therefore, as shown in Figure 11a, the collision time of the droplet is proportional to the Weber number, especially when the Weber number is larger. When the Weber number (*We*) increases, the collision complex of two droplets needs more time to recover to a spherical droplet. Therefore, the increase in coalescence time is not only due to the increased collision time, but also the recovery time. Moreover, according to the conclusions of Yuan et al. [16], at the same collision velocity, the larger the initial diameter of the droplet is, the more easily the collision complex breaks. Therefore, in the water-oil separation process, it is recommended to use small-diameter orifice plates for water-oil separation. When the specific gravity of the oil phase is 0.963, the minimum diameter of the droplet can be set to 0.2 mm. 

While the droplets approach each other, the oil film between the droplets produces a film thinning force. When the droplets collide head-on, the kinetic energy of the droplets overcomes the film thinning force with the highest efficiency. As the collision parameter increases, the efficiency of the droplets against the film thinning force gradually decreases. Therefore, the resistance of the droplets to approaching each other increases and the collision time increases. In addition, when the collision parameter increases, the loss of kinetic energy decreases, and the less kinetic energy is converted into surface energy. Therefore, as shown in Figure 11b, the recovery time and coalescence time increase with the increase of the droplet collision parameter. However, the effect of lower collision parameter on collision time and coalescence time is not obvious. Moreover, according to the comparison of the data in Figure 11a, it can be found that under the same Weber numbers (*We*), the coalescence time required for the off-center collision is longer than that for the center collision. Therefore, in water-oil separation, off-center collision due to stirring should be avoided.

## 5. Conclusions

In this study, the volume of fluid (VOF) method and adaptive mesh refinement (AMR) technology were used to study the collision and coalescence phenomenon of two water droplets in the transformer oil based on small amounts of polymer. The accuracy of the model was verified using experimental data. The grid independence of the model was also verified. Systematic comparative studies of the effects of initial droplet diameter and the collision parameter on droplet coalescence were presented. The simulations were carried out for a range of initial droplet diameters from 0.2 to 0.4 mm and collision parameter from 0.25 to 0.75. The following conclusions can be drawn from the results:

(1) The effect of initial droplet diameter on droplet coalescence. Droplet coalescence has a similar deformation process under the action of three initial droplet diameters, including radial stretching, radial contraction and axial contraction. As the initial droplet diameter decreases, the degree of droplet deformation decreases. When the initial droplet diameter is smaller (D_0_ = 0.2 mm), it takes less time for the droplet to coalesce and recover to a spherical droplet.

(2) The effect of collision parameter on droplet coalescence. (i) The collision process of the droplets presents different deformation modes with the increase of collision parameter under the same Weber number. The degree of droplet deformation is proportional to the collision parameter. The maximum axial diameter of the droplet at B = 0.75 is around 127% of the maximum axial diameter of the droplet at B = 0.25. (ii) The velocity flow field distribution in the coalescence process shows that with the increase of the collision parameter, the velocity vectors on both sides of the droplet become denser, which promotes droplet deformation.

(3) The droplet coalescence time is proportional to the initial droplet diameter and collision parameter. (i) As the initial droplet diameter increases, the Weber number increases. The collision time is proportional to the Weber number. When the Weber number is higher (*We* = 81.79), since the droplet takes longer to deform, the proportion of recovery time in the coalescence time increases. (ii) Under the same Weber number, the coalescence time of off-center collision is longer than that of a head-on collision. With the increase of the collision parameter, the efficiency of the droplets against the film thinning force gradually decreases, which leads to the increase of collision time and coalescence time. Therefore, in the water-oil separation, a smaller diameter orifice plate should be used. When the specific gravity of the oil phase is 0.963, the minimum diameter of the droplet can be set to 0.2 mm. In addition, stirring of the water-oil mixture should be avoided to shorten the time for droplet coalescence.

In the transformer oil based on small amounts of polymer, smaller initial droplet diameters and collision parameter are more conducive to coalescence. Therefore, in order to improve the efficiency of water-oil separation, it is recommended that the fluid flow uniformly through the smaller diameter orifice to produce the center collision of smaller droplets.

## Figures and Tables

**Table 1:** The setup of the numerical simulations.

Case	Initial Droplet Diameter *D_0_* (mm)	The Collision Parameter (*B*)	The Weber Number (*We*)	The Reynolds Number (*Re*)
A	0.2	0	40.89	50.80
B	0.3	0	61.34	76.20
C	0.4	0	81.79	101.60
D	0.4	0.25	81.79	101.60
E	0.4	0.5	81.79	101.60
F	0.4	0.75	81.79	101.60
